# Investigating patients with an immigration background in Canada: relationships between individual immigrant attitudes, the doctor-patient relationship, and health outcomes

**DOI:** 10.1186/s12889-016-2695-8

**Published:** 2016-01-12

**Authors:** Amanda Whittal, Sonia Lippke

**Affiliations:** 1 Bremen International School of Social Sciences, Jacobs University, Campus Ring 1, South Hall, 28759 Bremen, Germany; 2 McGill University, St. Mary’s Hospital, 3830 Lacombe Avenue, Montreal, H3T 1 M5 QC Canada; 3 Jacobs University Bremen, Campus Ring 1, 28759 Bremen, Germany

**Keywords:** Doctor-immigrant patient relationship, Doctor-immigrant patient communication, Acculturation orientation, Immigrant perceived quality of care, Immigrant health behaviours, Immigrant quality of life

## Abstract

**Background:**

Increasing immigration in the world today leads to more intercultural interactions. This is a particularly crucial fact in doctor-patient relationships, which often become more complex and suboptimal within an intercultural context. Since acculturation is a particularly important factor in this process, and the doctor-patient relationship is a key component in patient health outcomes, this study investigates the interrelation of individual immigrant acculturation orientations with the quality of the doctor-immigrant patient relationship, the patients’ perceived quality of care, and how this relates to immigrant health behaviours and quality of life of the patients.

**Methods:**

171 immigrant patients of various backgrounds participated in a paper and pencil questionnaire to assess the role of acculturation orientations (AO) on patients’ perceived expectations of their doctor, perceived quality of care (PQOC), health behaviours and quality of life. Data were analyzed using ANOVA, regression and correlation procedures with SPSS statistical software.

**Results:**

Significant correlations were found between all AOs and measures of the participant feeling connected to the host or home culture, thereby verifying the measure of AO. All four AOs were significantly interrelated directly with the patient’s perception of what the doctor expects of him/her, and the patients’ quality of life. Patients’ perceived expectations of their doctors were significantly related to the patients’ PQOC, and PQOC was associated with improved health behaviours (adherence to doctor recommendations, physical activity maintenance self-efficacy).

**Conclusions:**

AO may be an important factor in the doctor-immigrant patient relationship, via a complex process involving the patients’ perceptions of doctors’ expectations and perceived quality of care. This has important implications, since such an understanding can be used to create interventions for both doctors and immigrant patients to learn about their own AO, how it can relate to the quality of their relationship, and ultimately, the quality of care, health and quality of life of the patient.

**Electronic supplementary material:**

The online version of this article (doi:10.1186/s12889-016-2695-8) contains supplementary material, which is available to authorized users.

## Background

As socially interactive beings, our various relationships with other people/other groups of people play a substantial role in influencing our behaviours and experiences [1, 2]. These relationships are not limited to immediate family, friends, or even people of the same cultural background. According to the United Nations Population Fund (UNFPA), in 2010, 3 % of the world’s population (roughly 214 million people) lived somewhere other than their country of origin [3], a number that is expected to continue rising in many countries [4, 5]. Consequently, immigrants are becoming a more common part of many societies, and often play a valuable role in maintaining a strong workforce and economy of the countries to which they migrate [6]. As a consequence of this increasing globalization, our repertoire of relationships grows in scope, including people from a wide variety of different cultures. Such a widening of interactions has the potential to both enhance and hamper the life experiences of individuals in all facets of life.

One particular area in which this expansion of cultural interactions is crucial, is the doctor-patient relationship. Even without cultural factors, the relationship between doctors and their patients in notoriously complex and often suboptimal [7, 8]. It is subject to the influence of time pressure and stress commonly experienced by doctors [9, 10], to socioeconomic status [11], and to gender [12], to name a few. Because the doctor-patient relationship is a key element in successful health outcomes and patient satisfaction [13], several approaches have investigated this issue and how to improve it [14, 15]. This has led to a shift in Western thinking in the past few decades from an emphasis on a paternalistic doctor-patient relationship, toward one of a more equal partnership between doctor and patient [16]. Supporting this, there is a growing interest in the literature in patients’ perceptions of doctor-patient communication [11].

With more doctors treating immigrant patients due to the presently high migration rates, the doctor-patient relationship becomes even more complex, less optimal than relationships between doctors and native individuals, and poorly understood [17, 18]. Moreover, immigrants have been found to generally show poorer health behaviours and quality of life than the native population [19], to report lower quality of care [20] and lower quality of life [19] than the native population, and often do not adhere to their doctor’s medical advice [10, 21, 22].

Such negative outcomes for immigrant patients as those listed above have important individual and societal consequences, making this an essential area for investigation. On the individual and societal level, it has been found that since immigrants tend to be in poorer health, this may lead to higher unemployment or sick leave than the native population [23], which prevents the immigrant from being a fully functioning member of society. On a societal level, increased costs are incurred by the healthcare system when more people remain unwell due to ineffective visits with medical professionals. To consider the country of focus in this project, Canada, for example, has been experiencing an approximate $5,000,000 increase in healthcare costs per year since 2009 [24], and 19.8 % of the population had a migration background in 2006, which is expected to reach 29–32 % by 2031 [4].

Clearly, it is in the best interest of individuals and society as a whole to seek optimal health for citizens. Immigrant status, the doctor-patient relationship, and patients’ perceptions of doctors’ expectations are key elements that may influence the health of patients, but it is not well understood what role they play and how to address them when seeking to improve the doctor-patient relationship, and resulting health outcomes. This study therefore focuses on investigating how these factors interrelate with the health behaviours and quality of life of immigrant patients. Culture, from the point of view of an individual’s attitudes, may be an important contributor when investigating the fore mentioned factors.

One might ask what culture is: it is a vague term that is difficult to define and measure. Since an individual’s attitude (in this case, toward the culture from which they emigrated and the culture to which they came) can provide a fundamental basis on which interaction is built, culture is examined in this study from the perspective of acculturation orientations (AOs), based on Berry’s acculturation model [25]. This model is well established, and provides a functional method for operationalizing one’s cultural ‘attitude’, since it classifies an individual’s *acculturation orientation* when moving to a new ‘host’ culture into four categories: Assimilation – immigrant chooses to abandon own ‘home’ culture and adopts all ways and customs of the new ‘host’ culture. Integration – immigrant chooses to maintain some customs of ‘home’ culture, and also adopt some customs of ‘host’ culture. Separation – immigrant chooses to maintain all customs of ‘home’ culture, and refuses to adopt customs of ‘host’ culture. Marginalization – immigrant refuses to maintain customs of ‘home’ culture, or adopt customs of ‘host’ culture (see Additional file 1 for full model).

From the point of view of acculturation orientations, this research seeks to explore whether cultural attitudes (AOs) can provide an improved understanding of the relationship between doctors and immigrant patients, and the resulting health behaviours and quality of life of the immigrant patients. Specifically, it proposes the following hypotheses:

- H1: patient *acculturation orientation* (AO) interrelates with immigrant patients’ perceived expectations of their doctor on both a correlational and mean level.
- H2: patient AO predicts *patients‘ perceived quality of care* (PQOC), *health behaviours* (HB) and *quality of life* (QoL) after controlling for socio-demographic variables.
- H3: PQOC interrelates with patients’ HBs and QoL.

## Methods

This study adopts a quantitative methodology, with a focus on an immigrant patient population.

### Population

*N =* 171 immigrant patients (*M* = 54.38 years, *SD* = 17.94, Range = 23–96, 74.3 % female) of various backgrounds participated in a paper and pencil survey to assess the role of AOs on patients’ perceived expectations of their doctor, perceived quality of care, health behaviours and quality of life. The inclusion criteria required patients to have a migration background (immigrated to Canada after the age of 16), and be at least 18 years of age. Participant characteristics are outlined in Table 1.

**Table 1:** Participant characteristics

	Total (*n* = 171)
Number of men (%)	44 (26.0)
Mean age (SD), range	54.4 (17.9), 23–96
Marital status	
Single (%)	10 (5.9)
Close relationship, not living together (%)	3 (1.8)
Close relationship, living together (%)	1 (.6)
Married/in common law relationship (%)	129 (75.9)
Divorced (%)	12 (7.1)
Widowed (%)	15 (8.8)
Occupational Status	
Employed, full time (%)	61(35.7)
Employed, part time (%)	12 (7.0)
Student/ in training (%)	7 (4.1)
Unemployed/Job searching (%)	10 (5.8)
In pension/retired (%)	53 (31.0)
Housewife/husband (%)	18 (10.5)
Other (%)	10 (5.8)
Education	
None (yet) (%)	2 (1.2)
Primary School (%)	11 (6.4)
Secondary School (%)	8 (4.7)
High School (%)	23 (13.5)
Junior College (%)	27 (15.8)
University or Above (%)	95 (55.6)
Other (%)	5 (2.9)
Home culture	
Western Europe (%)	11(6.4)
Asia (%)	73 (42.2)
Mediterranean (%)	23 (13.3)
Africa (%)	15 (8.7)
America/Australia/New Zealand (%)	17 (9.8)
Eastern Europe (%)	24(13.9)

### Procedure

#### Recruitment

The study received ethical approval from the ethics committee at St. Mary’s Hospital, in Montreal, Canada, and the researcher was granted access to the hospital under a strict confidentiality agreement. Family doctors from St. Mary’s Hospital were invited face-to-face by the researcher to provide lists of their immigrant patients who fit the inclusion criteria.

After lists of immigrant patients were obtained, invitations to patients for participation followed the well-established ’Tailored Design Method’[26], in order to maximize response rate. The process was as follows: First, 330 patients were mailed invitation letters signed by their doctor, asking the patient to take part in a paper and pencil survey that would be mailed to their home, to help improve understanding about culture and health. The researcher followed up the mailed invitations with a phone call to the patients a few days later, ensuring permission to mail the surveys. Upon verbal agreement of the patient giving informed consent, surveys were mailed exactly one week after the invitation letter, including a $2 Canadian incentive. The rationale for mailing the incentive before completion of the survey was based on the reciprocity principle, which suggests that such an action increases the feeling of obligation for the potential participant to fulfil the request [26]. If within 7 days the completed survey was not returned, exactly one week from the survey mailing date, a thank-you/reminder letter was mailed, thanking the patient for their participation, and reminding them to participate if they had not already. This process of recruitment and survey mailing resulted in 171 returned completed surveys; a final response rate of 52 %, and acceptable level for hard to reach populations such as these. Further, as mentioned by Dillman et al., a response rate to mailed surveys between 50–70 % is considered successful [26].

All participants were invited, not required to take part in the survey, thus participation was voluntary. All potential participants were also informed that all information would be kept confidential.

### Measures

The questionnaire completed by the patients was nine pages, with the first two pages designed to determine their AOs. The questionnaire further assessed the patients’ health behaviours in terms of physical activity, nutrition, and adherence to medical advice; their perceived expectations of their doctors, their perceived quality of care received by their doctors, their perceived quality of life, and demographic characteristics.

According to the literature, AO is a rather difficult construct to measure [27, 28]. As a result, there are many, usually culture-specific, measurement tools available, which tend to target specific facets of AO, such as identity, behaviour and adaptation [29]. In order to create a more rounded measure, this study combined some of the better-established measurement tools available: the AO scale was comprised of three behaviour items from the general ethnicity questionnaire [30], one item on communication from the sociocultural adaptation scale [31], and three identity items [32] adapted from their original measure. All of the final items referred to the patients’ orientations towards the host culture (Canada), including such items as “it is important to me to see myself as Canadian”, and towards their home culture, including such items as “it is important to me to see myself as part of my home culture”[33]. All seven items were assessed for attitude toward the host culture (α = .71) and for attitude toward the home culture (α = .75), to calculate a single AO score for each individual.

The additional questions on the patient survey included items regarding individual stage of change of nutrition and physical activity behaviours (i.e. five possible answers to the question ‘Do you do physical activity for at least 2.5 h during the week, in a way that you are tired after?’ Possible answers were: ‘no and I do not intend to start’, ‘no but I am thinking about it’, ‘no but I seriously intend to start’, ‘yes but I only continued this for a short period of time’, ‘yes and I continued/will continue this for a long period of time’). Additionally, items regarding nutrition and physical activity self-efficacy were included (e.g. ‘I feel certain that I can be physically active for at least 2.5 h per week if…’ with answers on a four point scale from ‘completely disagree’ to ‘agree completely’) (α = .91) [34].

To assess quality of life, the 25-item World Health Organization quality of life questionnaire was used (α = .87) [35]. Perceived quality of care was assessed by six items, in response to the statement ‘my quality of care was…’ low/high, impersonal/personal, etc. Answer options ranged from zero to four (α = .96) [36]. Adherence to medical advice was assessed by five self -report items, which included statements such as ‘the last time I saw my doctor…it was hard to do what the doctor recommended I do’. Four possible answers ranged from ‘none of the time’ to ‘all of the time.’(α = .71 for positive items, α = .76 for negative items) [37].

All patient surveys were alpha-numerically coded to maintain confidentiality. Patients were also given the option to provide their contact information and be informed about final study results.

### Analytical strategy

All statistical analyses were run using IBM SPSS 20.

AOs were calculated for each individual patient using Euclidean Distance, a method proposed by Arends-Tóth&Van de Vijver [27]. Rather than placing individuals solely into one of the four categories, this method plots an individual’s scores on a two dimensional matrix, placing them where they are closest in orientation toward both the host culture and their culture of origin (i.e., full assimilation, etc.). Thus, instead of being categorized into one orientation, people are placed with proximity scores toward all of the orientations, enabling observation of which orientation they lean toward most. An example calculation can be seen in Additional file 2.

AO calculations revealed that the majority of participants leaned primarily toward the Marginalization category (*n* = 88: 52 % of sample), followed by the Separation category (*n* = 41: 24 % of sample), the Assimilation category (*n* = 22:13 % of sample) and finally, the Integration category (*n* = 17: 10 % of sample). Reliability analyses of these items had acceptable Cronbach’s Alpha scores for both the orientation toward the culture of origin (α = .75), and toward the host Culture (α = .71).

Total numbers of participants in each orientation category were calculated, and validity of the items was assessed by checking correlations of each orientation with the items ‘How strongly do you feel part of the Canadian culture?’ and ‘How strongly do you feel part of your home culture?’ (answers ranged on a four point scale from ‘not at all’ to ‘fully’).

To test H1 (patient acculturation orientation (AO) interrelates with immigrant patients’ perceived expectations of their doctor on both a correlational and mean level), a two-fold approach was used: first, correlation analyses were performed between each AO, and patients’ perceived expectations of their doctor. To investigate the formed groups in terms of the perceived expectations by controlling for sociodemographic variables, an ANCOVA was then run, with each AO as the independent variable, patients’ perceived expectations of their doctor as the dependent variable, and education, gender and age as covariates.

To test H2 (patient AO predicts patients’ perceived quality of care (PQOC), health behaviours (HB) and quality of life (QoL)), linear regression was used to test the interrelation of AO with PQOC, while controlling for the potential confounders age, gender and education. Correlation analyses were then used to investigate the relationship between AO, HB and QoL.

Finally, to test H3 (PQOC interrelates with patients’ HBs and QoL), linear regression was used with PQOC as the independent variable, and HB and QoL as the dependent variables.

## Results

### Acculturation orientation

Significant positive and negative correlations were found between all AOs and measures of the participant feeling connected to the host or home culture (Table 2), thereby verifying the AO measures. As expected, those leaning toward a Marginalization orientation felt significantly disconnected from both the host culture and culture of origin; those leaning toward a Separation orientation felt significantly less connected to the host culture and significantly more connected to the culture of origin; those leaning toward an Integration orientation felt significantly connected to both the host culture and the culture of origin; and those leaning toward an Assimilation orientation felt significantly more connected to the host culture, and significantly less connected the culture of origin (see Table 2).

**Table 2:** Correlations between AO and feeling connected to the host culture or culture of origin

	Marginalization	Separation	Integration	Assimilation
	(*N* = 83–85)	(*N* = 40–41)	(*N* = 15–17)	(*N* = 21)
Immigrant patient feeling connected to the host culture.	r = −.245^a^	r = −.277^a^	r = .279^a^	r = .437^a^
	Marginalization	Separation	Integration	Assimilation
Immigrant patient feeling connected to the culture of origin.	r = −.300^a^	r = .315^a^	r = .260^a^	r = −.145

^a^Correlation significant at .01 level

### Acculturation orientation and perception of Doctor’s expectations

Testing of H1 (patient acculturation orientation (AO) interrelates with immigrant patients’ perceived expectations of their doctor) revealed significant correlations between all four AOs and the patient’s perception of what the doctor expects of him/her. In particular, Marginalization was negatively correlated with both the perception that the doctor expects the immigrant patient to become part of the Canadian culture (*r* = −.363, *p* < .001), and the perception that the doctor accepts if the patient wants to keep his/her home culture (*r* = −.297, *p* < .001). In contrast, Separation was positively associated with the perception that the doctor accepts if the patient wants to keep his/her home culture (r = .202, *p* < .01). Further, Assimilation was positively associated with the perception that the doctor expects the patient to become part of the Canadian culture (*r* = .242, *p* < .01), and Integration was positively correlated with both the perception that the doctor expects the patient to become part of the Canadian culture (r = .362, *p* < .001), and the perception that the doctor accepts if the patient wants to keep his/her home culture (*r* = .242, *p* < .01).

**Fig. 1:**
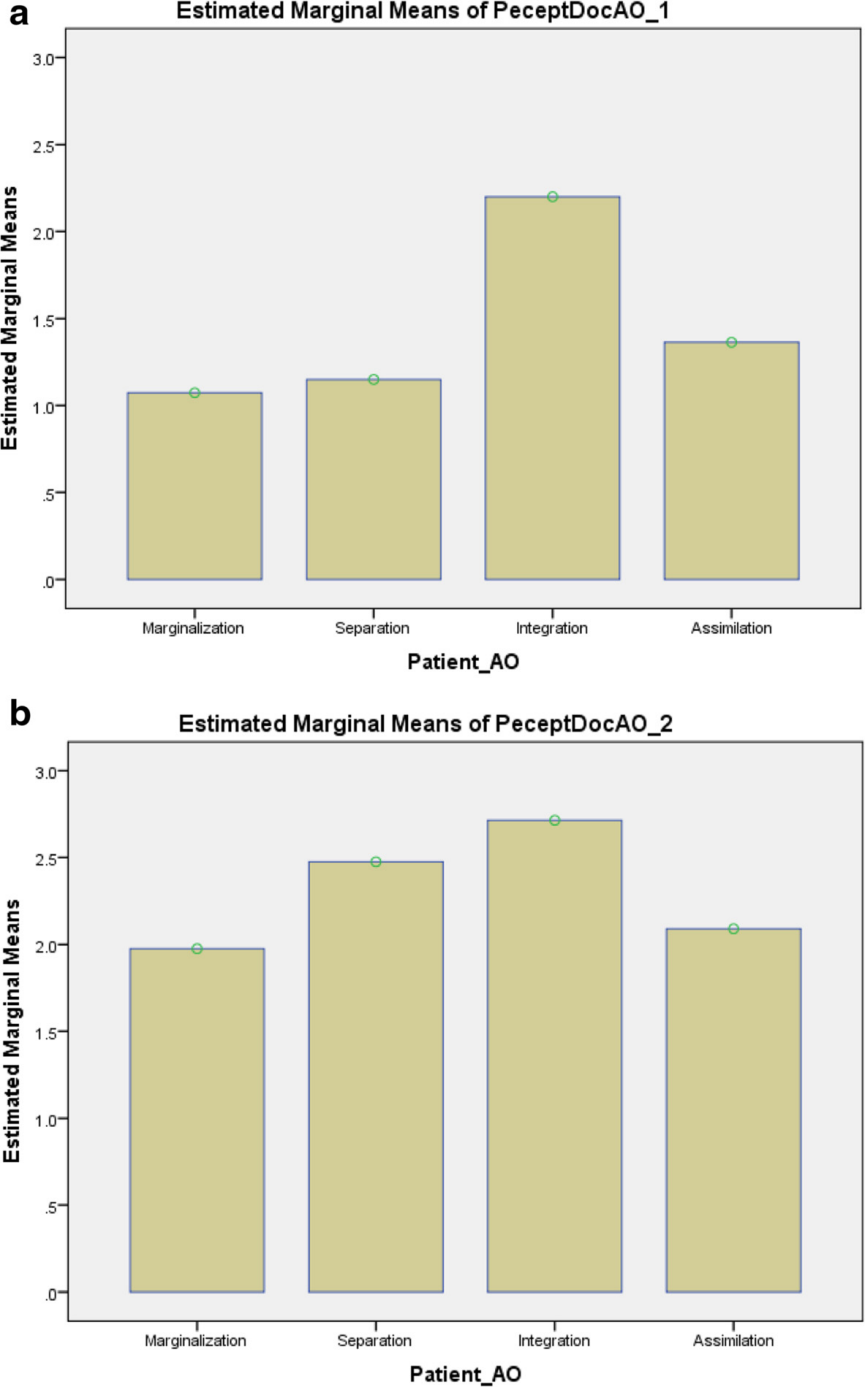
**a** AO and immigrants’ perception that the doctor expects them to become part of the Canadian culture. **b** AO and immigrants’ perception that the doctor accepts if they want to keep their home culture

To investigate the formed groups in terms of perceived expectations, while controlling for potential covariates (gender, age and education), the association between AO and the patients’ perception of what the doctor expects of him/her was tested with a one-way ANCOVA. AO accounted for a significant amount of the variance for both the perception that the doctor expects the patient to become part of the Canadian culture (*F*(3, 148) = 4.567, *p* < .01, partial η^2^ = .085), and the perception that the doctor accepts if the patient wants to keep his/her home culture (*F*(3, 149) = 4.081, *p* < .01, partial η^2^ = .076) when controlling for the three covariates (see Fig. 1a and b).

Of the covariates, neither gender nor age played a significant role in accounting for variance of either dependent variable. Only education contributed more substantially to the perception that the doctor expects the patient to become part of the Canadian culture, although it was only borderline significant (*F*(1, 148) = 3.765, p = .054). Effect sizes, calculated in terms of partial Eta squared (partial η^2^), were negligible for all covariates as compared to AO (< .01). Results are displayed in Tables 3 and 4.

**Table 3:** AO and immigrants’ perception that the doctor expects them to become part of the Canadian culture

Variables	df	F (mean square)	Partial Eta Squared (η^2^)	*p*
AO	3	4.567 (4.538)	.085	.004*
Age	1	1.349 (1.341)	.009	.247
Gender	1	.007 (.007)	.000	.935
Education	1	3.765 (3.741)	.025	.054

* = significant at the *p* < 0.01 level. Covariates: age, gender and education

**Table 4:** AO and immigrants’ perception that the doctor the doctor accepts if they want to keep their home culture

Variables	df	F (mean square)	Partial Eta Squared (η^2^)	*p*
AO	3	4.081 (3.778)	.076	.008*
Age	1	.300 (.277)	.002	.585
Gender	1	.106 (.098)	.001	.745
Education	1	.823 (.762)	.005	.366

* = significant at the *p* < 0.01 level. Covariates: age, gender and education

### Acculturation orientation and perceived quality of care

For the first part of H2 (patient AO predicts patients’ perceived quality of care (PQOC)), linear regression revealed that AO did not have a significant influence on patients’ PQOC directly. The perception that the doctor accepts if the patient wants to keep his/her home culture was, however, significantly positively related to the patients’ PQOC when controlling for age, gender and education (*b = .22, t*(2.76), *p* < 0.01, R^2^ = .056).

### Acculturation orientation, health behaviours and quality of life

Testing the second part of H2 (patient AO predicts patients’ health behaviours (HB) and quality of life (QoL)), linear regression revealed that AO could not predict HBs or QoL, however, weaker but significant correlations were found between AO and reported quality of life. Reported quality of life was negatively correlated with Marginalization (r = −.215, *p* < .01), but positively correlated with Integration (r = .210, *p* < .01). Similar strength significant correlations were also found between AO and health behaviours, with Assimilation positively correlated with physical activity motivational self-efficacy (*r* = .204, *p* < .010) and physical activity stage of change (rho = .171, *p* < .05); and Separation negatively correlated with physical activity motivational self-efficacy (*r* = −.192, *p* < .05), physical activity maintenance self-efficacy (*r* = −.177, *p* < .05), and physical activity stage of change (rho = −.219, *p* < .01).

Lastly, in testing H3 (PQOC interrelates with patients’ HBs and QoL), linear regression revealed that higher PQOC was associated with improved adherence to doctors’ recommendations (*b = .19, t*(2.754), *p* < 0.01, R^2^ = .046), and improved physical activity maintenance self-efficacy (*b = .19, t*(2.42), *p* < 0.05, R^2^ = .029) after controlling for age, gender and education. The revealed relation between PQOC and adherence to doctors’ recommendations is noteworthy, as neither AO nor patients’ perceived expectations of doctors related to this crucial health behaviour.

## Discussion

This study investigated the relationship between immigrant acculturation orientation (AO) and their perceived expectations of their doctors, perceived quality of care, health behaviours and quality of life in 171patients with a migration background. Since past literature has found that individuals’ AOs interrelates with their perceptions and behaviours [38, 39], it was expected that this is also true within the domain of health. Further, patient’s perceptions of doctors’ expectations have been found to be interrelated with the doctor-patient relationship [40], which can in turn influence the quality of care and, health behaviours and quality of life of the patient [41].

It was first tested whether the measurements of AO showed acceptable reliability. Significant correlations were found between all four AOs and measures of the participant feeling connected to the host or home culture. These results suggest that our measures of AO accurately reflect how connected or disconnected an immigrant feels to his or her home or host culture. This is important, as it means that the measured constructs provide an important indication about immigrants’ basic *attitudes* toward their home and host cultures.

H1 posited that patient AO interrelates with immigrant patients’ perceived expectations of their doctor. Significant correlations were found between all four AOs and the patient’s perception of what the doctor expects of him/her. Specifically, those with a Marginalization orientation perceived that their doctor had little or no expectations that the patient should adjust to the new culture (Canada), or maintain their previous culture. Those with a Separation attitude were more likely to perceive that the doctor accepts if they want to maintain their previous culture, and less likely to perceive that the doctor expects them to adapt to the Canadian culture. Those with an integration attitude were more likely both to perceive that the doctor accepts if they want to maintain their previous culture, and that the doctor expects them to adapt to the Canadian culture. Finally, those with an Assimilation attitude were less likely to perceive that the doctor accepts if they want to maintain their previous culture, and more likely to perceive that the doctor expects them to adapt to the Canadian culture. This partially replicates and adds to previous research asserting that individual AO can affect the individual’s perceptions [38, 39]. These findings seem to suggest that AO may indeed affect individuals’ perceptions, which may provide some insight into why they behave in certain ways. It could also be that the doctors’ attitudes play a role in this perception, and should be taken into account in future studies.

H1 was therefore supported: AO significantly interrelates with patients’ perceived expectations of their doctor. As mentioned previously, patients’ perceived expectations of their doctor can play an important role in shaping the doctor-patient relationship [11, 40]. Thus, the interrelation between AO and patients’ perceived expectations of their doctor is an important factor to be considered when assessing the quality of a doctor-patient relationship which, as mentioned, tends to be both complex and suboptimal [8]. It is important to mention that these patients did not choose their doctor, so the association is most likely an influence of the patient’s own perceptions and attitudes.

H2 suggested that patient’s AO predicts patients’ perceived quality of care (PQOC), health behaviours and quality of life (QoL). Regression results revealed that AO itself could not predict patients’ PQOC. However, patients’ perceptionsof doctors’ expectations could. In particular, the perception that the doctor accepts if the patient wants to maintain his or her own culture was related to an increase in patients’ PQOC. This is an important finding, as it shows the potential of AO as a factor in patients’ PQOC, by influencing patients’ perceptions of their doctor’s expectations. It is also a relevant result in that immigrant patients tend to report lower PQOC than the native population [19], and often do not adhere to their doctor’s medical advice [10, 21, 22]. Understanding the factors that influence PQOC can lead to steps to improve this. In this case, it may be that AO acts through a more indirect pathway and therefore does not directly relate to PQOC, but rather relates with the perceptions of doctors’ expectations, which seems to then relate to PQOC. Perceived expectations of the doctor that match with the patients’ own level of comfort and safety may be important for the patient experiencing a high PQOC.

In terms of reported health behaviours and QoL, AO was found to be significantly related to both. The Assimilation orientation was related to improved physical activity motivational and physical activity stage of change, indicating that individuals of this orientation are more likely to have higher physical activity self-efficacy, and more likely to be in a more active stage of change for physical activity. Moreover the Separation orientation was related to poorer physical activity motivational self-efficacy, physical activity maintenance self-efficacy, and physical activity stage of change, indicating that individuals of this orientation are more likely to have lower physical activity self-efficacy, and more likely to be in a stage of engaged physical activity. These findings support previous research, which has found and posited that integration into a new society may be beneficial, while separation from the new society may be detrimental for immigrants [42]. This may be explained by the fact that integration into a new society makes it easier for one to engage in healthy lifestyle behaviours like physical activity, which could require a certain level of functioning within society if, for example, a person wants to attend a gym.

The Marginalization orientation correlated with lower general QoL, while the Integration orientation correlated with higher general QoL. These results provide some support for the notion that when becoming part of a new culture, the Assimilation and Integration orientations are probably more beneficial for the immigrant than the Marginalization and Separation orientations. This could again possibly be explained by the assimilation/integration orientations allowing for a certain ease of functioning within a society on levels of daily life, communication and socializing, all of which are related to quality of life. Translating this into public health approaches, it suggests the potential importance of facilitating integration of immigrants into their new country, through publicly available activities, trainings or seminars. Since these results come from correlational data they can only point to possibilities, not causal relations. In order to make this potential more certain, further research with more predictive value, preferably longitudinal, is necessary. The notion that interventions are needed to improve immigrant integration has been noted on numerous occasions in past literature [14, 15].

H2 was therefore partially supported: AO could not directly PQOC, but patients’ perceived expectations of doctors is significantly interrelated with PQOC. AO interrelated with only *some* aspects of Health Behaviours, and did interrelate with QoL. AO could not, however, account for medical advice adherence or other health behaviours. These findings also reflect the findings of previous research, which has found that perceived expectations of doctors interrelate with PQOC [40].

The above findings were further illuminated with investigation into H3. H3 suggested, based on previous literature findings that PQOC may be influential in patient’s HBs and QoL [20], that PQOC interrelates with patients’ HBs and QoL.

PQOC did in fact relate to some health behaviours, most importantly, medical advice adherence.

The complex nature of these findings in total support previous research, which has found that the doctor-immigrant patient relationship is a key element in health outcomes and patient satisfaction, and needs to be improved [13]. There are, however, a multitude of factors influencing it, making it a far from straightforward process [14, 15]. This may also provide some insight as to the reasons the correlations on many variables, while significant, were on the weaker side. This could suggest that AO does play an important role, but it remains speculative at this point as to where it fits among other important factors on the PQOC, HBs and QoL of immigrant patients, a question yet to be disentangled.

The results of this paper therefore provide some initial evidence for the important role of immigrant patients’ AOs in doctor-patient relationships, and the potential health related outcomes. The findings suggest that AO directly interrelates with patients’ perceptions of their doctors’ expectations (H1), patients’ QoL, and some health behaviours (H2). Further, there may be a potential mediation relationship, since AO could not significantly relate to patients’ PQOC, but patients’ perceptions of their doctors’ expectations (which was significantly related to AO) did significantly interrelate withPQOC. PQOC in turn, interrelated with additional aspects of patients’ self-efficacy and health behaviours. This point is crucial, as PQOC related to a health behaviour that AO showed no direct relationship with: medical advice adherence. Previous research has found that immigrant patients show poorer medical advice adherence than patients native to a culture [10, 21, 22]. Therefore, a better understanding of how to potentially improve such behaviour would be extremely beneficial. This is an important call for future research to test the influence of AO in other countries and contexts, and different immigrant groups, in an effort to develop a solid foundation on which interventions can be based.

The results presented in this study are far from concretely answering the question of how acculturation orientations are related to the doctor-patient relationship, health behaviours and quality of life. They do, however, provide some initial guidance into a domain that could have immense potential in providing a foundation on which to improve doctor-immigrant patient relationships, health and quality of life of the patients. In explaining such findings, their potential becomes more salient.

As mentioned, this is a complex process. One factor not assessed here, but which may also potentially be playing a role, is the AO of the doctors. While this study has chosen to make an initial start by examining only the perspective of the patient, the AO of the doctor and the resulting interaction is another important and possibly influential factor to be addressed.

### Limitations

Although this study contributes important information, some limitations should be noted. Firstly, the data were cross-sectional, so causal effects cannot be claimed. Future studies should seek to collect longitudinal data on this subject, to establish more concrete causal relations.

Secondly, although the sample size was sufficiently large for the analyses conducted, for stronger and more robust statistical tests and results, larger, more representative samples, and from different countries, would be needed.

We did not assess peoples’ motivations for migration, which could certainly have some influence on pressure and incentives to either maintain their previous culture, or adopt ways of the new culture. Looking at such distinctions is yet another important aspect, and could open an additional body of research.

The use of self-report data further comes with its own host of problems, in that it is dependent on accurate assessment and recording by the participants. Replications of the research design could seek ways of collecting objective data - at least for health behaviours - in addition to self-report measures. Any future studies using the survey method should consider the fact that different cultural ideas about health and quality of life can affect survey response. It is hoped that this issue was at least partly avoided in this study through clarity of explanation in the construction of each survey question, but should nonetheless always be considered.

Lastly, in relation to the first point of this section, to further test this theory as an empirical model, more advanced statistical analysis techniques could be applied. This is particularly relevant, since there is some basis on which to hypothesize that there could be a mediation relationship between AO, perceptions of doctors’ expectations, PQOC, HBs and QoL.

## Conclusion

Despite the fore mentioned limitations, this study was able to examine the role of immigrant patient acculturation orientation in the doctor-patient relationship, and the resulting health behaviours and quality of life of the patient. The evidence provided points toward AO as a potentially influential factor in the doctor patient relationship, via a complex process involving the patients’ perceptions of doctors’ expectations and perceived quality of care. These factors relate both directly and indirectly to the health behaviours and quality of life of immigrant patients. Future research could consider investigating the details of this process, including the influence of AO in other countries and contexts, with different immigrant groups, using longitudinal data, and more advanced analyses.

Since doctor-patient interactions tend to be more challenging with immigrant patients, and these same patients tend to report poorer health in general, health behaviours and quality of life, it is imperative to improve the relationship between doctors and immigrant patients. Having a solid evidence based foundation of knowledge can help to enhance understanding of this topic. A long term goal should be to create interventions for both doctors and immigrant patients to improve their relationship, the quality of care, health and quality of life of the patient. A better understanding of AOs may be a good starting point for providing important information and insights, to eventually reach such a goal.

## Additional files

Additional file 1: **Berry’s acculturation model.** (DOCX 14 kb)

Additional file 2: **Euclidean distance calculation example: patient A.** (DOCX 13 kb)

## References

[1] Knight CJ, Rodgers WM, Reade IL, Mrak JM, Hall CR. Coach Transitions: Influence of Interpersonal and Work Environment Factors. Sport, Exercise, and Performance Psychology. 2015. http://dx.doi.org/10.1037/spy0000036.

[2] Saita E, Acquati C, Kayser K. Coping with Early Stage Breast Cancer: Examining the Influence of Personality Traits and Interpersonal Closeness. Frontiers in Psychology. 2015. doi: 10.3389/fpsyg.2015.00088. 10.3389/fpsyg.2015.00088 PMC4318273 25699003

[3] United Nations Population Fund. 2014. http://www.unfpa.org/pds/migration.html.

[4] Malenfant EC, Lebel A, Martel L. Projections of the diversity of the Canadian population, 2006-2031 (Statistics Canada, Catalogue 91-551-X) Ottawa: Statistics Canada, 2010.

[5] DestatisStatistischesBundesamt. Migration & Integration Statistics. 2013. https://www.destatis.de/EN/FactsFigures/SocietyState/Population/Migration/Current.html

[6] Facchini G, Mayda A (2012). Individual Attitudes Towards Skilled Migration: An Empirical Analysis Across Countries. World Economy.

[7] Priebe S, Sandhu S, Dias S, Gaddini A, Greacen T, Ioannidis E (2011). Good practice in health care for migrants: views and experiences of care professionals in 16 European countries. BMC Public Health.

[8] Tarrant C,Windridge K, Baker R, Freeman G, Boulton M. Falling through gaps: primary care patients’ accounts of breakdowns in experienced continuity of care. Family Practice. 2014. doi:10.1093/fampra/cmu077. 10.1093/fampra/cmu077 PMC5926435 25411422

[9] Van Ryn M, Burke J (2000). The effect of patient race and socio-economic status on physicians’ perceptions of patients. Social Sciences & Medicine.

[10] Villagran M, Hajek C, Zhao X, Peterson E, Wittenberg-Leyles E. Communication and culture: Predictors of treatment adherence among Mexican immigrant patients. J Health Psychology. 2011. 1*–*10. doi:10.1177/13591053114171094. 10.1177/1359105311417194 21900335

[11] Verlinde E, De Laender N, De Maesschalk S, Deveugele M, Willems S. The social gradient in doctor-patient communication*.* International Journal for Equity in Health*.* 2012, 11(12). doi:10.1186/1475-9276-11-12. 10.1186/1475-9276-11-12 PMC3317830 22409902

[12] Schieber AC, Delpierre C, Lepage B, Afrite A, Pascal J, Cases C, et al. Do gender differences affect the doctor–patient interaction during consultations in general practice? Family Practice. 2014. doi:10.1093/fampra/cmu057. 10.1093/fampra/cmu057 25214508

[13] Shrivastava SR, Shrivastava PS, Ramasamy J (2014). Exploring the dimensions of doctor- patient relationship in clinical practice in hospital settings. Int J Health Policy Manag.

[14] Jagosh J, Boudreau JD, Steinert Y, MacDonald ME, Ingram L (2011). The importance of physician listening from the patients’ perspective: Enhancing diagnosis, healing, and the doctor–patient relationship. Patient Educ Couns.

[15] Rolfe A, Cash-Gibson L, Car J, Sheikh A, McKinstry B. Interventions for improving patients’ trust in doctors and groups of doctors. Cochrane Consumers and Communication Group. 2014. doi:10.1002/14651858.CD004134.pub3. 10.1002/14651858.CD004134.pub3 PMC7386923 24590693

[16] Davis JL (2008). Healthcare and Listening: A Relationship for Caring. Int J Listening.

[17] Dunn JR, Dyck I (2000). Social determinants of health in Canada’s immigrant population: results from the national population health survey. Social Sciences and Medicine.

[18] Marks DF (2002). Freedom, responsibility and power: Contrasting approaches to health psychology. J Health Psycholgy.

[19] Nesterko Y, Braehler E, Grande G, Glaesmer H (2013). Life satisfaction and health- related quality of life in immigrants and native-born Germans: the role of immigration- related factors. Qual Life Res.

[20] Saha S, Arbelaez JJ, Cooper LA (2003). Patient-Physician relationships and racial disparities inquality of health care. Am J Public Health.

[21] Harmsen H, Meeuweesen L, van Wieringen J, Bernsen R, Brudijnzeels M (2003). When cultures meet in general practice: intercultural differences between GPs and parents of child patients. Patient Educ Couns.

[22] Van Wieringen J, Harmsen J, Bruijnzeels M (2002). Intercultural communication in general practice. Eur J Public Health.

[23] Akhavan S, Bildt OC, Franzen EC, Wamala S (2004). Health in relation to unemployment and sick leave among immigrants in Sweden from a gender perspective. J Immigr Health.

[24] Statistics Canada. Health and social service institutions revenue and expenditures. 2013. http://www.statcan.gc.ca/tables-tableaux/sum-som/l01/cst01/govt32a-eng.htm.

[25] Dillman DA, Smyth JD, Christian LM (2009). Internet, mail and mixed-mode surveys: The tailored design method.

[26] Arends-Tóth J, Van de Vijver FJR. Assessment of psychological acculturation: Choices in designing an instrument. In: Sam DL, Berry JW, editors. Cambridge Handbook of Acculturation Psychology. Cambridge: Cambridge University Press; 2006a.

[27] Rudmin F (2009). Constructs, measurements and models of acculturation and acculturative stress. Int J Intercultural Relations.

[28] Taras, V. Instruments for measuring acculturation. 2008. Retrieved from http://ucalgary.ca/~taras/_private/Acculturation_Survey_Catalogue.pdf.

[29] Tsai JL, Ying Y-W, Lee PA (2000). The meaning of “being Chinese” and “being American”.Variations among Chinese American young adults. J Cross-Cult Psychol.

[30] Searle W, Ward C (1990). The prediction of psychological and sociocul-tural adjustment during cross-cultural transitions. Int J Intercultural Relations.

[31] Roccas S, Sagiv L, Schwartz S, Halevy N, Eidelson R (2008). Toward a unifying model of identification with groups: Integrating theoretical perspectives. Pers Soc Psychol Rev.

[32] Möllering A, Schiefer D, Knafo A, Boehnke K (2013). Acculturation and well-being among migrant and minority adolescents: a cross-national and cross-ethnic comparison. The challenges of diaspora migration: Interdisciplinary perspectives on Israel and Germany.

[33] Lippke S, Ziegelmann JP, Schwarzer R, Velicer WF (2009). Validity of stage assessment in the adoption and maintenance of physical activity and fruit and vegetable consumption. Health Psychol.

[34] World Health Organization (WHO). Programs: Mental Health Publications. 1991. http://www.who.int/mental_health/publications/whoqol/en/

[35] Richmond VP, Smith RS, Heisel AM, McCroskey JC (1998). The impact of communication apprehension and fear of talking with a physician and perceived medical outcomes. Commun Res Rep.

[36] Hays, R.D. The medical outcomes study (MOS) measure of patient adherence. 2002. Retrieved from http://www.rand.org/content/dam/rand/www/external/health/surveys_tools/mos/mos_adh erence_survey.pdf

[37] Montreuil A, Bourhis RY (2001). Majority acculturation orientations toward “valued” and “devalued” immigrants. J Cross-Cult Psychol.

[38] Van Leeuwen N, Rodgers RF, Bui E, Pirlot G, Chabrol H (2014). Relations between acculturation orientations and antisocial behavior in adolescents and young adults from immigrant families. Int J Culture and Mental Health.

[39] Babitsch B, Braun T, Borde T, David M (2008). Doctor’sperception of doctor-patient relationships in emergencydepartments: whatroles do gender and ethnicityplay?. BMC Health Serv Res.

[40] Swain S, Hariharan M, Rana S, Chivukula U, Thomas M (2015). Doctor-Patient Communication: Impact on Adherence and Prognosis Among Patients with Primary Hypertension. Psychol Stud.

[41] Levecque K, Van Rossem R (2015). Depression in Europe: does migrant integration have mental health payoffs? A cross-national comparison of 20 European countries. Ethn Health.

[42] Berry JW, Kazarian SS, Evans DR (1998). Acculturation and health: Theory and research. Cultural clinical psychology: theory, research and practice.

