# Supplementary material for: Investigating patients with an immigration background in Canada: relationships between individual immigrant attitudes, the doctor-patient relationship, and health outcomes
Source: BMC Public Health. 2016 Jan 12;16:23. doi: 10.1186/s12889-016-2695-8 (PMC4709992; doi:10.1186/s12889-016-2695-8)
Supplement: Supplementary file 1 — Berry’s acculturation model. (DOCX 14 kb) [file 12889_2016_2695_MOESM1_ESM.docx]

Appendix A

*Berry’s Acculturation Model*

**Cultural Maintenance (Of Immigrant OR Host Culture)**
 High Low

| **Integration** Interest in maintaining one’s original culture while also participating in daily and social activities of the dominant group and with other ethnic and cultural groups | **Assimilation** Individual does not wish to maintain his/her cultural identity and seeks daily interactions with other cultures |
| --- | --- |
| **Separation** Individuals place a high value on holding onto their original culture and avoid interaction with others | **Marginalization** Little possibility or interest in having relationships with others and little interest in or possibility of cultural maintenance (due primarily to experiences with discrimination or instituationalized, forced separation from others). |

High

**Contact and Participation

(Of
Immigrant OR
 Host Culture**


Low

(takenfromBerry, 1998)
