# Supplementary material for: Investigating patients with an immigration background in Canada: relationships between individual immigrant attitudes, the doctor-patient relationship, and health outcomes
Source: BMC Public Health. 2016 Jan 12;16:23. doi: 10.1186/s12889-016-2695-8 (PMC4709992; doi:10.1186/s12889-016-2695-8)
Supplement: Supplementary file 2 — Euclidean distance calculation example: patient A. (DOCX 13 kb) [file 12889_2016_2695_MOESM2_ESM.docx]

Appendix B – Euclidean Distance Calculation Example: Patient A

**Scores:**

Completely disagree = 1
Slightly disagree = 2
Slightly agree = 3
Agree completely = 4

**Mean of answers to questions about home culture:** 2.86

**Mean of answers to questions about host culture (Canada):** 2.43

**Calculate Distance Score from each orientation with Euclidean Distance Formula:**

D(x, y) = √ (x_1_ – y_1_)^2^ + (x_2_ – y_2_)^2^

X_1_ = mean score on questions about home culture

X _2_= mean score on questions about host culture

Y_1_ and Y_2_ = Most extreme scores for each orientation (Marginalization = 1, 1; Separation = 4, 1; Assimilation = 1, 4; Integration = 4, 4).

**Distance Scores for Patient A:**

From full Marginalization: √(2.86-1)^2^ + (2.43-1)^2^ = √5.5 = 2.35

From full Separation: √(2.86-4)^2^ + (2.43-1)^2^ = √3.34 = 1.83

From full Assimilation: √(2.86-1)^2^ + (2.43-4)^2^ = √5.92 = 2.43

From full Integration: √(2.86-4)^2^ + (2.43-4)^2^ = √3.76 = 1.93

***Distance scores can range from 0-4.24. Proximity scores to each orientation are then calculated:**

Marginalization: 4.24- 2.35 = 1.89

Separation: 4.24-1.83= 2.41

Assimilation: 4.24-2.43 = 1.81

Integration: 4.24-1.93 = 2.31

***These scores are used to plot the person in two dimensional space, and observe visually toward which orientation they lean the most***
